# Roles, function and relevance of LAG3 in HIV infection

**DOI:** 10.1371/journal.ppat.1007429

**Published:** 2019-01-17

**Authors:** Colin G. Graydon, Allison L. Balasko, Keith R. Fowke

**Affiliations:** 1 Department of Medical Microbiology and Infectious Diseases, University of Manitoba, Winnipeg, Canada; 2 Department of Community Health Sciences, University of Manitoba, Winnipeg, Canada; 3 Department of Medical Microbiology, University of Nairobi, Nairobi, Kenya; 4 Partners for Health and Development in Africa, Nairobi, Kenya; University of Alberta, CANADA

## Abstract

HIV causes several forms of immune dysfunction that need to be addressed in a functional cure for HIV. Immune exhaustion describes a dysfunctional phenotype caused by chronic cellular activation. Lymphocyte activation gene-3 (LAG3) is one of several negative coreceptors known as immune checkpoints that contribute to this exhaustion phenotype. Antibodies targeting immune checkpoints are now used clinically to restore immunity against cancer and hold promise in restoring immunity during HIV infection. Here, we summarize current knowledge surrounding LAG3 and discuss its relevance during HIV infection and the potential for LAG3-targeting antibodies in a functional HIV cure.

## Author summary

Antiviral drugs have transformed HIV infection from a death sentence to a manageable disease. However, millions still lack access to these drugs, which require daily adherence. This allows the HIV epidemic to continue, killing a million people each year. The search for a cure has been long, but researchers now have a greater knowledge of HIV and human biology than ever before and are designing drugs and strategies that may be used in a cure. Checkpoint blockade is one strategy already used to treat cancer but may also eventually be used in an HIV functional cure, which would allow the body to control HIV without the help of antiviral drugs. Checkpoint blockade works by inhibiting molecules called immune checkpoints, which are the brakes of the immune system. Here we focus on an immune checkpoint called LAG3. LAG3 can be present on many different immune cells with differing frequency but is more abundant during HIV, in which having more LAG3 is associated with certain aspects of worse disease. Although it may have slightly different functions on different types of cells, overall, LAG3 reduces the cell’s ability to respond to stimulus. Inhibiting LAG3 could reinvigorate immune cells to fight HIV and may even help fight coinfections such as hepatitis viruses. Inhibiting other immune checkpoints along with LAG3 may improve efficacy. If combined with other drugs and strategies to fight HIV, checkpoint blockade may allow the immune system to control HIV without the help of antiviral drugs—a functional cure.

## Introduction

Antiretroviral therapy (ART) inhibits human immunodeficiency virus (HIV) replication, but a reservoir of latently infected cells means that ART must be taken indefinitely and thus does not constitute a cure. The ideal HIV cure would completely eradicate HIV. However, a functional cure, in which HIV is permanently suppressed in latent reservoirs, is more feasible with lower cost and less severe side effects. Restorative immunotherapy may help achieve a functional cure by reversing the immune exhaustion during HIV infection.

Immune exhaustion describes a phenotype of misplaced tolerance coinciding with expression of inhibitory proteins, known as immune checkpoints (IC) (e.g., lymphocyte activation gene-3 [LAG3], programmed cell death-1 [PD1], TIM3 [T-cell immunoglobulin and mucin-domain containing-3], TIGIT [T-cell immunoreceptor with Ig and ITIM domains], CTLA-4 [cytotoxic T-lymphocyte-associated protein-4], BTLA [B- and T-lymphocyte attenuator], 2B4), that impair cellular immune response. Like other ICs, LAG3 likely evolved as an immuno-regulatory strategy to protect from organ damage during aberrant or excessive immune activation (e.g., allergy, autoimmunity, inflammatory bowel disease)[1–4]; however, when a strong immune response is desired, misplaced LAG3-mediated immunosuppression may be detrimental. Immune exhaustion harms are evident in cancer, in which antibodies blocking PD1 and CTLA-4 substantially increase survival and have become first-line treatment for advanced melanoma [5]. Indeed, the 2018 Nobel Prize in Physiology or Medicine was awarded to pioneers of this research [6]. Although ICs may seem redundant, their differing expression patterns and signaling mechanisms, and their functional synergy provide the opportunity to take advantage of functional redundancies to more accurately target and titrate immune restoration. For HIV, reversing immune exhaustion may restore immunity, thereby reducing opportunistic infection and improving control of HIV. Here, we review LAG3, its relevance in HIV infection, and its therapeutic potential within a functional cure.

## LAG3 expression

LAG3, a member of the immunoglobulin superfamily, is expressed on T cells, natural killer (NK) cells, plasmacytoid dendritic cells (pDCs) and B cells. LAG3 is frequently studied on T-cells, in which it translocates to lipid rafts on the cell surface after cellular activation, forming dimers and oligomers which colocalize with cluster of differentiation 3 [CD3] and CD4/CD8 upon reactivation [7–9]. T-cells LAG3-expression generally increases with differentiation [10–12].

The lymphocytic choriomeningitis virus (LCMV) infection mouse model is useful for studying LAG3 in vivo because acute and chronic strains exist. After 1 to 2 weeks of LCMV infection, LAG3 expression peaks on T-cells. In the acute model, virus is cleared and LAG3 expression decreases, allowing activated cells to differentiate into memory cells. In the chronic model, LAG3 remains elevated, representing exhaustion [13,14]. Then, like other ICs, LAG3 is elevated during HIV, cancer, tuberculosis, and hepatitis B and C [11,15–22]. This up-regulation is driven on T-cells by T-cell receptor (TCR) stimulation and on activated NK and T-cells by interleukin 12 [IL-12] in an interferon-γ [IFNγ]-dependent manner [23,24]. Other cytokines, such as IL-27, IL-15, IL-2, and IL-7, also up-regulate LAG3 [1,23–27]. LAG3 may also regulate and be regulated by T-bet (a T-box transcription factor), which along with Eomesodermin, guides differentiation of cytotoxic T lymphocytes (CTLs). Indeed, deletion of T-bet increases LAG3 expression on murine T-cells, and in turn LAG3 knockout increases T-bet [28,29]. HIV-specific CTLs are overwhelmingly T-bet^dim^, even following ART-initiation[30].

Although TCR stimulation and cytokines up-regulate LAG3, toll-like receptor (TLR) stimulation may oppose this, at least in mice, potentially by increasing expression and activity of transmembrane matrix metalloproteases A disintegrin and metalloproteinase domain-containing protein 10 [ADAM10] and ADAM17 [31], which regulate LAG3 through membrane cleavage [32], or by increasing T-bet activity [33]. During HIV infection, LAG3 remains elevated despite presence of TLR ligands. ART may reduce LAG3 on bulk T-cells but not on central memory T-cells, NKs, or invariant-NK T-cells (iNKTs) [10,11,15]. Whether this regulation of LAG3 is appropriate during HIV infection is unclear. However, PD1 blockade successes in HIV, discussed later, indicate that ICs are overexpressed [34].

## LAG3 receptors

Discovered in 1990, LAG3 was recognized to share ancestral homology and structural similarity to CD4 [35]. LAG3 regulates T-cell activation mainly through interaction with major histocompatibility complex (MHC) class II, binding with 100-fold the affinity of CD4 [35–38]. Unlike CD4, however, LAG3 does not bind the HIV envelope glycoprotein 120 [gp120], so it is thought not to be an HIV receptor [37]. However, as Human Leukocyte Antigen-DR (HLA-DR), an MHC-II molecule, is expressed on activated T-cells, both LAG3 and its main receptor are elevated during HIV infection, perhaps enhancing immunosuppression.

In mice, LAG3 also binds two lectins, Galectin-3 (Gal3) and liver and lymph node sinusoidal endothelial cell C-type lectin (LSECtin), in both cases suppressing CTL IFNγ production [39,40]. HIV and its trans-activator of transcription (Tat) protein up-regulate Gal3, which also enhances HIV budding and transfer [41,42]. Gal3 exists intracellularly and extracellularly with distinct functions and binds to many glycosylated proteins with diverse physiological outcomes [43]. In comparison, LSECtin is lesser known and participates in antigen recognition, uptake, and internalization [44]. Any evolutionary reason for the interaction between these lectins and LAG3 is unclear but likely dependent on the heavy glycosylation of LAG3. Research is needed to confirm LAG3–lectin interaction in humans to determine the importance of Gal3 and LSECtin.

## LAG3 mechanism

LAG3 differs from other ICs because it lacks noticeable inhibitory motifs. Early studies of LAG3’s mechanism supported the idea that LAG3 inhibited T-cell activation through competition with CD4—just as CTLA-4 competes with CD28 for CD80/86 [45], by demonstrating that LAG3 inhibited IL-2 production of a CD4^+^ T-cell hybridoma but not of a CD4^−^ variant—and that this inhibition depended on MHC-II [46]. However, this same study showed that mutating a lysine residue in the cytoplasmic domain abolished LAG3 function, implying an intracellular mechanism rather than receptor competition. This is further supported by evidence that LAG3 inhibits CTLs (discussed below), has non-MHC-II ligands [11], binds a different site of MHC-II than CD4, and does not competitively inhibit CD4 during TCR ligation [38]. A potential explanation for this apparent paradox is that LAG3 inhibits intracellular signaling proteins dependent on CD4 or CD8, such as lymphocyte-specific protein tyrosine kinase (Lck). Details of LAG3’s intracellular mechanism remain uncharacterized except that LAG3 inhibits calcium flux and nuclear factor of activated T-cells (NFAT) activation during TCR stimulation [47,48]. Although a LAG3-associated protein binds the glutamine proline (EP) motif of LAG3’s intracellular domain, deletion of this motif did not abrogate LAG3’s function [46,49]. Research of LAG3’s role and mechanism has largely been restricted to inhibition of TCR-dependent stimulation and its interaction with MHC-II. However, the antibody typically used to inhibit murine LAG3 (clone C9B7W) does not block the LAG3 MHC-II interaction [50]. Furthermore, LAG3 maintains its role on non–T-cells, as discussed below. Therefore, LAG3 may also function in TCR-independent stimulations such as through cytokine or pattern recognition receptors. Therefore, more research into the LAG3 mechanism and situational roles is critical.

## LAG3 function on cell types and subsets

### LAG3 on conventional T-cells

**Fig 1:**
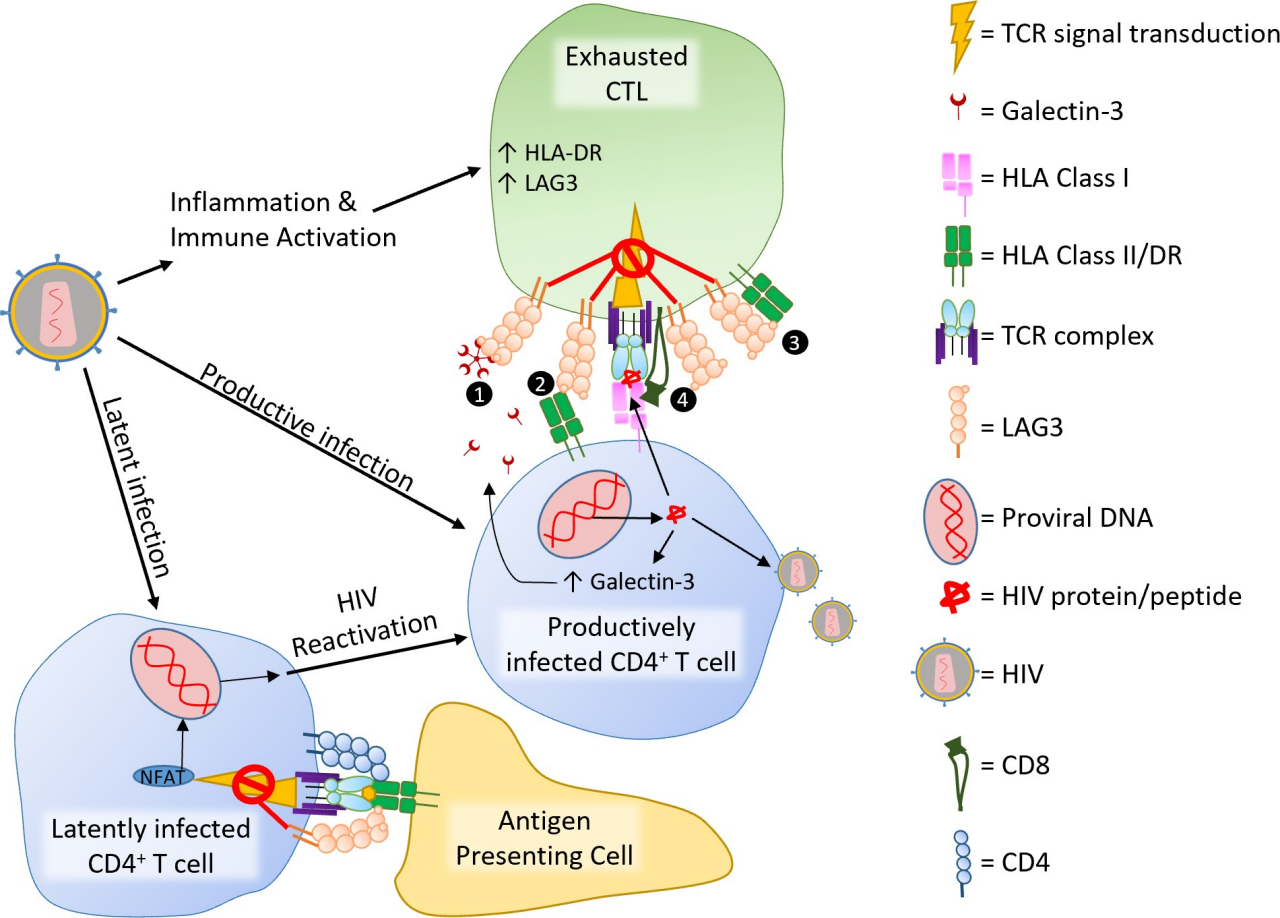
LAG3 on T-cells during HIV infection. HIV induces inflammation and immune activation, which leads to exhausted lymphocytes and increased expression of LAG3 and HLA-DR, an HLA class II molecule. LAG3 may inhibit CTL activation by binding to (1) the alternative LAG3 ligands LSECtin or Gal3, (2) HLA class II molecules that traffic to the immune synapse on the target cell or (3) HLA class II molecules that traffic to the immune synapse on the same cell, or by (4) cotrafficking with CD8 to the immune synapse, which occurs during CD8 crosslinking [109]. Productive HIV infection increases production of Gal3, which can inhibit CTL activation and killing. Productive infection can be induced from latent infection through activation of certain transcription factors, including NFAT, which is inhibited by LAG3 during T-cell activation. CD, cluster of differentiation; CTL, cytotoxic T lymphocytes; Gal3, Galectin-3; HLA, human leukocyte antigen; LAG3, lymphocyte activation gene-3; LSECtin, liver and lymph node sinusoidal endothelial cell C-type lectin; NFAT, nuclear factor of activated T-cells; TCR, T-cell receptor.

T-cell exhaustion is generally viewed as CTL dysfunction, but CD4^+^ T-cell exhaustion also exists [51]. Although cell-line studies show that LAG3 inhibits CD4^+^ T-cell function [11,46,47], differential function of LAG3 between these cells and CTLs remains largely uninvestigated. Although one study found no effect of LAG3-blockade on CTL activation [52] and another study demonstrated that inhibition of CTL function is dependent on CD4^+^ T-cell LAG3 expression[53], others show that LAG3 inhibits CTL independently of CD4^+^ T-cells [22,54]. Overall, studies suggest LAG3 regulates the CTL response but may have greater impact when expressed on CD4^+^ T-cells. LAG3-inhibition of CTLs is curious because MHC-II, which CTLs do not recognize, is the main receptor for LAG3. We propose several possible mechanisms for this curiosity (Fig 1). Although LAG3-mediated regulation protects T-cells from activation-induced cell death following intense stimulation, LAG3 offers no protection from apoptosis following physiologically relevant stimulation [7,14,55].

### LAG3 on T regulatory cells

Whether T regulatory cells (Tregs) are beneficial or detrimental in HIV pathogenesis is unclear. As previously reviewed, Tregs abate HIV-related inflammation but also suppress desired anti-HIV immunity [56–58]. Likewise, Treg LAG3 expression is higher than for conventional T-cells [59,60]; its role on Tregs remains controversial. Early studies suggested LAG3 enhances Treg function when the inflammatory and/or antigenic burden is high [1,59] but not in less demanding environments [53,59,61], in which LAG3 expressed on the responding T-cell had greater impact [29,53]. In contrast, studies using similar models show LAG3 inhibiting Treg function and impairing Treg development [29,62,63]. One consistent finding throughout these studies is that LAG3 inhibits proliferation of Tregs. The literature is similarly unclear on LAG3’s role in Treg inhibition of dendritic cell (DC) maturation, with studies showing conflicting results [60,64]. In summary, LAG3’s role on Tregs is not well defined but seems dependent on immune microenvironment.

### LAG3 on plasmacytoid dendritic cells

As important producers of type I IFN and links between innate and adaptive immunity, pDCs play a significant role during HIV infection. However, like Tregs, the beneficial or detrimental effects of pDCs on HIV disease progression is debated. On one hand, pDCs may help control HIV by inhibiting viral replication and promoting CTL and NK cells through IFNα production and cross-presentation, especially during early infection [65]. Yet, pDC-mediated immune activation may also contribute to immunopathology and immune exhaustion [65].

In mice, pDCs express 10-fold greater LAG3 than Treg and conventional T-cells [66]. LAG3-expressing pDCs behave more tolerogenically than LAG3-negative pDCs—fewer IFNα, more IL-6, increased generation of Tregs, and induction of monocytes to recruit myeloid-derived suppressor cells [17]. Furthermore, LAG3 on pDCs inhibits T-cell expansion and vice versa [66]. These immunosuppressive and *trans*-cellular LAG3 effects may explain why LAG3 knockout mice have increased numbers of cell types that naturally lack LAG3—including granulocytes and macrophages [67]—suggesting that LAG3 inhibits expansion of other innate immune cells by encouraging a suppressive environment.

### LAG3 on NK and innate T-cells

LAG3 knockout mice have increased numbers of innate cells that would otherwise express LAG3, including γδT cells, NKs, and pDCs, implying that it regulates their expansion [67]. LAG3 expression on innate cells—including mucosal-associated invariant T-cells (MAIT; 20%)[68], NKs and iNKTs (1%–15%) [15,16], pDC (6%) [17], and γδT-cells (19% in mice) [50]—is higher compared to conventional T-cells.

An early report showed LAG3-knockout or -blockade enhancing murine-NK killing of tumor cell lines [69]; however, a contrasting study was unable to confirm this in human NKs [70]. Recently, two murine cancer models showed that LAG3-blockade after IL-12 administration reduced metastases and increased number and functionality of NKs, implying a similar regulatory role for LAG3 on NKs as on T-cells [71]. Because LAG3 is up-regulated on NKs during HIV infection and is higher on those of HIV progressors compared to HIV controllers, this regulatory role represents a potential source of NK dysfunction during disease [16].

Our laboratory has shown LAG3 to be up-regulated on iNKTs during HIV infection [15,16]. iNKTs are innate-like cells, with both NK and T-cell markers, that respond to lipid antigens presented by the MHC-I–like molecule CD1d and are important linkers of the innate and adaptive immune system [72]. Although beneficial for anti-HIV immunity, iNKTs are depleted and functionally impaired during HIV infection [73]. We have previously shown that LAG3, but not PD1, expression on iNKTs in HIV-infected individuals is inversely correlated with their ability to produce IFNγ [15]. Furthermore, LAG3 inhibits proliferation of iNKTs [74]. Together, this indicates that LAG3 acts as an IC on iNKTs similarly to T-cells.

MAITs are an innate T-cell subset that comprise 1%–10% of peripheral T-cells but are exhausted and depleted from the periphery during HIV [75]. Although their antiviral function is not clear, MAITs respond to bacterial metabolites and are likely important in defence against bacterial coinfections during HIV [75]. Recently, it was discovered that LAG3 is highly up-regulated on MAITs following activation, causing functional impairment that is reversible with LAG3-blockade [76]. MAIT LAG3 expression during HIV is unknown, but LAG3-blockade may partially reverse their dysfunction during disease.

Innate T-cells are more abundant in the gut and liver than peripheral blood. These are sites of intense inflammation during HIV infection, suggesting LAG3 expression in these areas may be even greater than in the blood. HIV can cause liver disease [77], in which iNKTs and MAITs comprise up to 30% and 50% of lymphocytes, respectively [78,79], and the LAG3 ligand LSECtin is highly expressed [80]. During hepatitis B and C infections, LAG3 is up-regulated on T-cells, in which it reduces cytokine production and cytotoxicity but is uninvestigated on MAITs, NKs, and iNKTs [20–22]. Taken together, these studies indicate LAG3 is of great importance in the liver during HIV, hepatitis B virus (HBV), and hepatitis C virus (HCV) infection. Due to complex immune environments and severe immune-mediated liver damage often caused by these phasic infections, further study is needed to determine whether LAG3 is harmful or beneficial.

Overall, LAG3 is understudied on innate cells. This is especially true of γδT-cells. γδT-cells expressing Vδ2 are dysfunctional and severely depleted from peripheral blood during HIV infection [81]. Although LAG3 expression on γδT-cells has been noted [35,82], little research has investigated its expression or function in these cells.

## LAG3 and HIV disease

Immune exhaustion is a main facet of immune dysfunction and is associated with poor HIV disease outcomes. Indeed, LAG3 is associated with high viral load [11,19,83], faster disease progression [19], and rapid return of viraemia following treatment interruption [83]. Moreover, LAG3 is down-regulated on NKs of HIV elite controllers—individuals with undetectable viral load despite being infected—and HIV-exposed seronegative (HESN) populations, and CD4^+^ T-cells of viraemic nonprogressors and elite controllers [16,84,85]. These studies do not resolve cause from effect, because LAG3 may represent immune activation rather than contribute to disease progression, but they demonstrate that LAG3 is associated with unfavourable disease measurements and could be a main contributor to immune exhaustion in HIV. Reversing immune exhaustion may restore immunity against coinfections and enhance HIV-specific immunity, making it a candidate for use in a functional cure.

### LAG3-blockade: Potential in an HIV functional cure

The functional HIV cure goal is to suppress virus in latent reservoirs, eliminating the need for ART. Elite controllers demonstrate that such control is possible. A “shock and kill” tactic, in which “shock” refers to reactivation of HIV from latent reservoirs and “kill” denotes depletion of these now visibly infected cells, is one immune-based strategy for a functional cure. LAG3-blockade could be one component of this strategy by reversing latency and simultaneously enhancing HIV-specific immunity (Fig 1).

### LAG3 and HIV latency

LAG3 is elevated in lymph nodes and tissues, areas in which the HIV reservoir is prominent [11]. Because activation of NFAT and nuclear factor kappa-light-chain-enhancer of activated B cells (NF-κB) promotes HIV-transcription [86] and LAG3 inhibits NFAT and cellular activation, its blockade may reverse latency [47]. Furthermore, memory T-cells expressing LAG3 alone, or coexpressing LAG3 with TIGIT and PD1, respectively, harbor 2-fold and 8-fold more integrated HIV DNA than their negative counterparts, implying an LAG3 or IC combination blockade would preferentially target infected cells [12]. Although studies have not investigated whether LAG3 helps maintain HIV latency, studies have shown that PD1-blockade enhances latency reversal and exposes HIV in latently infected cells to the adaptive immune system for potential elimination [87–91].

### Checkpoint blockade enhances HIV immunity

Strong immunity is critical to the “kill” aspect of the “shock and kill” tactic. Moreover, CTLs are necessary to maintain control of HIV [92]. Reversing immune exhaustion could effectively restore this immunity. Indeed, only a short duration of LAG3-blockade enhances the formation of memory T-cells during viral infections [14,93].

Although LAG3 expression is not elevated on gag-responsive CTL compared to cytomegalovirus CMV-responsive counterparts, PD1 expression is [10,94]. As an approved first-line treatment for advanced melanoma, research into PD1-blockade is more developed than for LAG3-blockade. Although cells expressing abundant PD1 may be irreversibly exhausted, LAG3 is typically expressed without PD1 during HIV [11,12,95]. Furthermore, PD1-blockade improves frequency and response of HIV-specific CTL, and reduces viral load and mortality in simian immunodeficiency virus-infected macaques, indicating exhaustion in HIV is reversible [90,96]. Promising PD1-blockade studies in humanized mice [97] and ex vivo [94] have resulted in early clinical trial attempts to improve anti-HIV immunity. Thus far, case reports demonstrate increases in cell-associated HIV RNA and reductions in latent reservoir size after PD1/CTLA-4–blockade [89,98,99], and one clinical trial showed improved HIV-specific CTL responses in a subset of individuals taking PD-L1–blockade [100]. However, retinal toxicity in a parallel macaque model study led to this human trial being stopped. This adverse event slowed IC-blockade research for HIV and warrants caution, although a recent similar study witnessed no side effects [90]. It is unclear whether IC-blockade for HIV is any less safe than for cancer; however, HIV necessitates a different risk–benefit calculus for IC-blockade compared to cancer, with similar risk but lower benefit considering the alternative of lifelong ART. LAG3-blockade may be relatively safe because LAG3 knockout mice do not readily exhibit immunopathology, in contrast to PD1 and CTLA-4 deficient mice [101–103]. Indeed, preliminary trial results show LAG3+PD1 combination blockade has a similar safety profile to PD1 monotherapy in advanced melanoma patients [104]. Furthermore, when PD1 is blocked, LAG3 and other ICs are often up-regulated in compensation and vice versa [62,105,106]. Therefore, although PD1-blockade is promising for HIV cure, other ICs or an IC combination blockade may improve safety or efficacy. Indeed, animal models of chronic viral infection and cancer have demonstrated that PD1+LAG3 combination blockade is more effective than either alone [107,108], and of the 33 ongoing registered clinical trials of LAG3-blockade, all but two are testing LAG3+PD1 combination blockade. Preliminary results of one such clinical trial demonstrates striking efficacy of LAG3+PD1-blockade (BMS-986016 and nivolumab, respectively) for treatment of advanced melanoma patients whom PD1/PD-L1-blockade previously failed (16% objective response rate and 45% disease control rate) [104].

## Conclusion

Despite LAG3’s importance in cancer, allergy, autoimmunity, and infectious disease, much about its function is undefined. Although mechanisms and functions of LAG3 remain controversial, LAG3 clearly inhibits immune responses. Therefore, a main concern surrounding IC-blockade for HIV is that it may increase inflammation and immune activation, thereby accelerating disease. Although a valid concern, the preliminary successes of PD1-blockade support the argument that reversing immune exhaustion will result in favorable outcomes. Regardless, LAG3 and other IC-blockades should be investigated in HIV models with and without viral suppression to understand their roles during disease.

**Fig 2:**
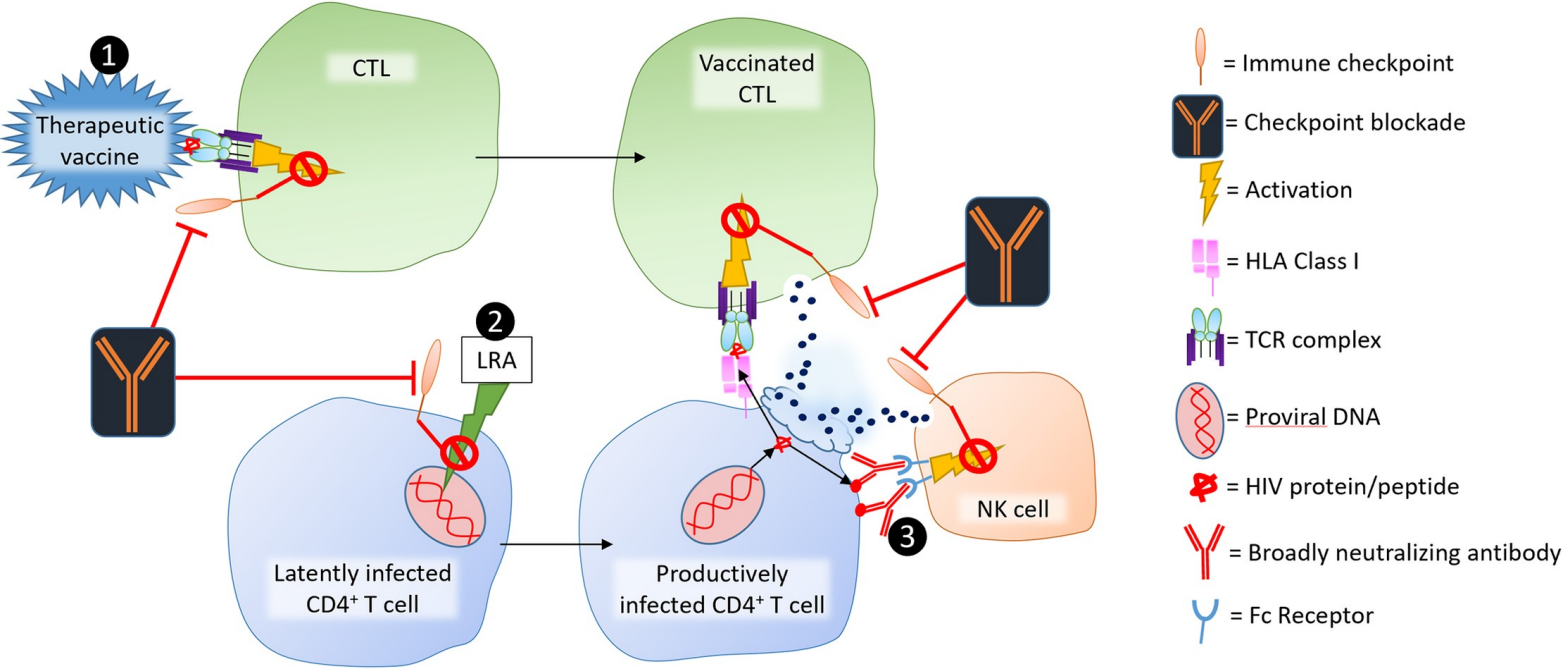
Potential role for checkpoint blockade in combination immunotherapy for a functional cure. A therapeutic vaccine (1) would enhance HIV-specific CTL number and function. Because many HIV-specific CTLs are exhausted during HIV, IC blockade could enhance the activating effect of the vaccine and the function of the CTL after activation. After administering this vaccine, checkpoint blockade could feasibly enhance LRAs (2) activity as previously demonstrated for PD1 [91]. During LRA treatment, broadly neutralizing antibodies (3) could bind to HIV polypeptide expressed on the infected cell’s surface and activate antibody dependent cell cytotoxicity activity by NK cells, which may also be enhanced by checkpoint blockade [110]. CD4, cluster of differentiation 4; CTL, cytotoxic T lymphocyte; HLA, human leukocyte antigen; IC, immune checkpoint; LRA, latency reversing agent; NK, natural killer; PD1, programmed cell death-1; TCR, T-cell receptor.

If LAG3-blockade improves immune function during HIV infection, it could help deplete the HIV reservoir by reversing latency and restoring immunity of exhausted cells. Providing ART is a priority; therefore checkpoint inhibition should be pursued for individuals with ART-suppressed virus. For untreated individuals, checkpoint blockade may increase cellular production of and susceptibility to HIV by enhancing immune activation. Furthermore, replicating HIV can evolve under selective pressure to evade CTL responses, an issue resolved by ART. However, few HIV antigens are exposed when HIV is suppressed. Therefore, during viral suppression, IC-blockades would likely be most effective when combined with triggers or other immunotherapies (Fig 2). Immunotherapies like IC-blockade have advantages, including almost no risk of HIV resistance and potentially improved broad immunity, particularly against coinfections. These advantages and the potential for a functional cure justify cautious optimism and further research of LAG3 expression, mechanism, and function.
